# A cross-over feasibility trial of smartphone grayscale mode in medical students

**DOI:** 10.3389/fdgth.2026.1816095

**Published:** 2026-07-06

**Authors:** James Hagerty, Shrey Saretha, Brenton Phung, Van Le, Jebgy Vargas, Fouzia Khan, Deborah Wright

**Affiliations:** Department of Medical Education, The California University of Science and Medicine, Colton, CA, United States

**Keywords:** digital behavior change, digital distraction, grayscale mode, medical students, screen time, smartphone

## Abstract

**Background:**

Smartphones are central to digital health environments, yet their color-rich interfaces may increase visual salience and habitual engagement. Grayscale mode removes color saturation, potentially reducing attentional capture during digital task use.

**Methods:**

We conducted a cross-over feasibility trial involving first-year medical students at an allopathic medical school. Participants were assigned in a 1:1 alternating sequence based on order of enrollment to use either grayscale screen settings or standard color display for one week, followed by a crossover to the alternate condition for a second week. Participants recorded daily screen time using their smartphone's built-in tracking feature and completed end-of-week surveys assessing attitudes regarding sleep quality and productivity. Linear mixed-effects models were used to analyze differences in screen time between conditions.

**Results:**

Fifty-one participants completed the study. Grayscale mode was associated with a statistically significant reduction in mean daily screen time compared to color display (mean reduction 28 min per day; *p* < 0.05). The effect was consistent across intervention order. Qualitative comments suggested reduced habitual engagement alongside usability challenges for certain tasks.

**Conclusions:**

Grayscale mode represents a low-cost, scalable interface-level intervention associated with reduced smartphone screen time. These findings provide preliminary evidence that modifying visual features of widely available consumer technology may influence digital engagement patterns in high-demand learning environments.

## Introduction

Smartphones are central to modern learning and digital health environments, yet increasing screen time has been associated with distractibility, reduced self-control, and adverse mental health outcomes among students (1–4). Visual salience plays a critical role in attentional capture. According to color theory, high contrast and saturated colors increase visual engagement. Experimental work has demonstrated that color influences eye movement and fixation patterns, suggesting that interface design can shape attentional allocation (5).

Although prior grayscale intervention studies have been conducted in college and pharmacy student populations (6, 7), medical students may represent a distinct high-exposure learner group because smartphone use is embedded in academic tasks, clinical communication, scheduling, rapid information access, and mobile study resources. Medical students also rely on smartphones for medical education, communication, medical applications, and rapid access to information during training (8). In this context, evaluating grayscale mode among medical students is not simply a replication in another student population, but an assessment of whether an interface-level intervention remains feasible and potentially effective in a training environment where smartphone use may be both necessary and distracting.

This cross-over feasibility trial evaluates whether enabling grayscale mode on smartphones reduces screen time among medical students. By modifying visual salience at the interface level, this study examines a scalable digital behavior-change strategy embedded within existing consumer technology.

## Methods

### Study design

This cross-over feasibility trial was conducted over two consecutive weeks. A 1-week period per condition was selected to balance feasibility and participant burden while allowing sufficient time for stabilization of routine smartphone use under each display condition.

Participants were assigned in a 1:1 alternating sequence based on order of enrollment to maintain balance between groups. Allocation was performed prior to study initiation. Due to the visible nature of the grayscale display intervention, participants were not blinded to condition; however, the cross-over design allowed each participant to serve as their own control.

During the first phase of the study, participants in Group A were instructed to change their smartphone display settings to grayscale mode, while Group B maintained their usual color display settings. Over the course of the week, participants were instructed to record their daily screen time, as reported by their smartphone's built-in screen time tracking feature.

At the end of each study week, participants completed an anonymous survey in which they reported daily screen time for each day of the preceding week. Instructions were provided to ensure consistent access to device-specific tracking features. The survey also included structured self-report items assessing perceived study habits, productivity, and sleep quality, along with an optional open-ended free-text section to capture qualitative experiences. Survey items were developed by the study team for exploratory purposes and were not derived from a validated instrument.

Following this first week, participants crossed over to the alternate condition, with Group A returning to standard color display and Group B switching to grayscale mode. At the end of the second week, all participants completed the same survey administered at the end of the first week. No washout period was implemented between phases because grayscale mode represents an interface-level visual modification with no anticipated physiological carryover effects once the display setting was changed. Behavioral carryover effects, such as habit formation, were considered less likely because the intervention duration was likely too short to establish a lasting pattern of behavior (9).

Participants were asked to self-report screen time without screenshot verification and were instructed to record 0 min if data were unavailable for a given day. Operating system-specific instructions were provided for both iOS and Android users, including disabling cross-device tracking features where applicable to ensure consistency in measurement.

Sleep quality and study habits were assessed using brief descriptive self-report items intended for exploratory contextual interpretation rather than as primary outcome measures. Screen time was defined as the primary outcome, while survey-based measures of productivity and sleep quality were considered secondary exploratory outcomes.

Feasibility outcomes included recruitment, retention, adherence to the assigned display condition (assessed via self-report), and acceptability, assessed through qualitative free-text responses. This cross-over design allowed each participant to serve as their own control, thereby minimizing between-subject variability and improving internal validity.

### Participants and recruitment

First-year medical students at an allopathic medical school were invited via institutional email and posted flyers to attend a study information session.

At the beginning of the information session, all participants were presented with an informed consent form detailing the purpose of the study and how the collected data would be used for research purposes. Participants received detailed instructions on how to enable grayscale mode on their smartphones, how to view their daily screen time using their device's built-in tracking features, and how to complete the post-intervention surveys. In exchange for their participation, participants were compensated for each weekly survey they completed.

### Inclusion and exclusion criteria

Participants in this study were first-year medical students at the California University of Science and Medicine. Inclusion criteria required participants to be enrolled in their first-year of medical school, attend the research study informational session, and possess a smartphone with screen time tracking capabilities. Exclusion criteria included individuals without access to a smartphone, those with visual impairments including color blindness that could interfere with the grayscale intervention, and those with diagnosed neurocognitive or psychiatric conditions affecting sleep or attention. Students who were unable to attend the information session, declined to provide informed consent, were unwilling to modify their phone settings or were unable to complete the required surveys were also excluded from participation.

### Attrition

**Figure 1:**
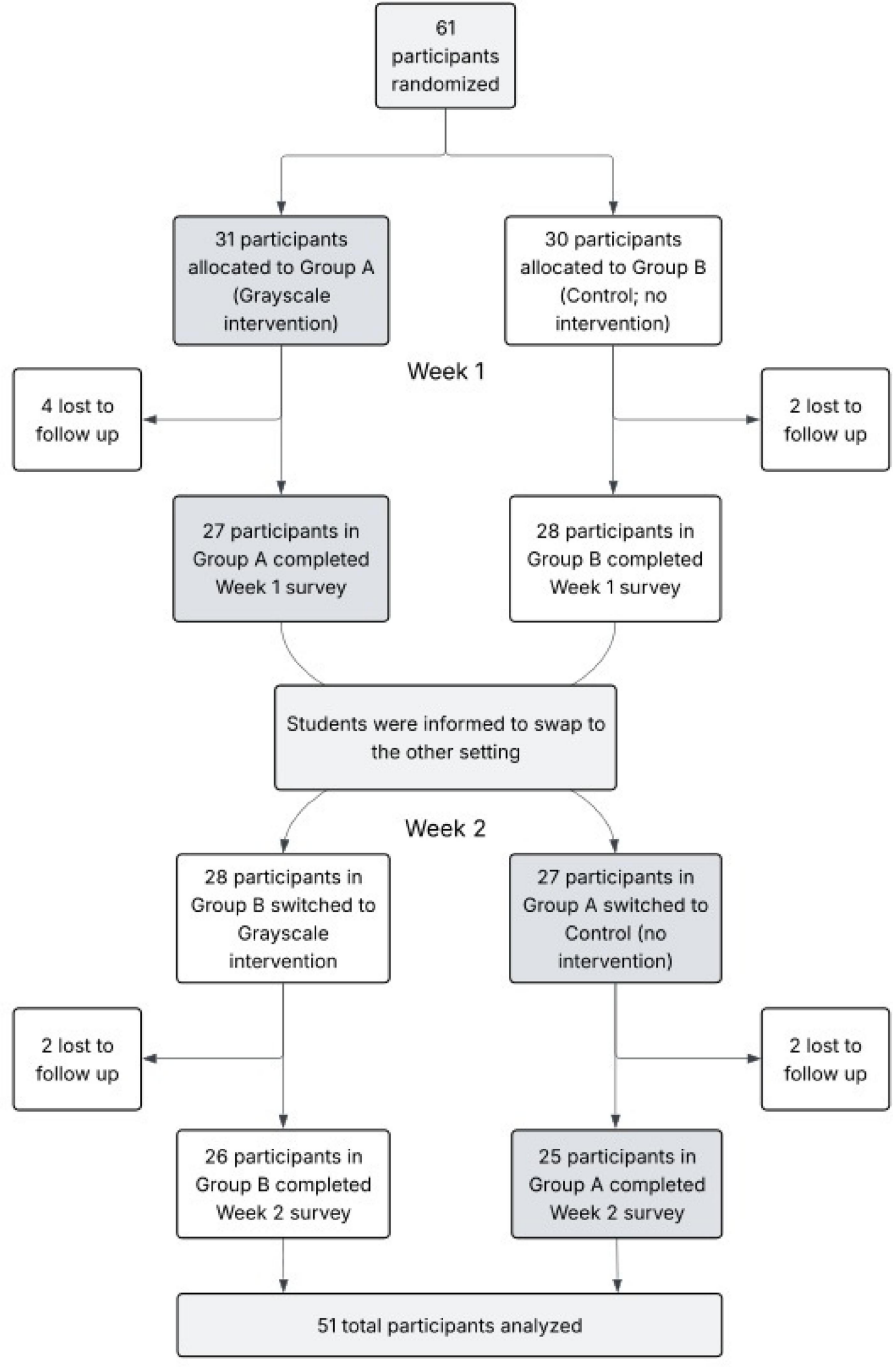
Participant flowchart.

Loss to follow-up was defined as non-response at the time of survey distribution. Participant flow through the study is summarized in Figure 1.

### Statistical methods

No *a priori* power calculation was performed; the study was designed as a feasibility and exploratory trial based on the available cohort of first-year medical students. Mean, standard deviation, minimum, and maximum values were calculated for each sequence group (Group A vs. Group B) and condition (grayscale vs. control). Individual daily screen time observations were used as repeated measures in the mixed-effects analyses. For the primary analysis, linear mixed-effects models were fitted with condition (grayscale vs. control) as a fixed effect and participant as a random intercept to account for repeated measures within individuals. Restricted maximum likelihood (REML) estimation was used, and statistical inference was based on Satterthwaite's approximation of degrees of freedom. Model assumptions were assessed through residual diagnostics.

In addition, a pooled comparison of screen time across all participants by condition was conducted. For this exploratory analysis, mean daily screen time values were averaged within each condition, and differences between grayscale and control were evaluated using a paired-samples t-test.

All statistical analyses were conducted in R, with two-sided *p*-values < 0.05 considered statistically significant.

Qualitative responses were analyzed thematically. Free-text comments were reviewed, coded inductively, and organized into domains reflecting perceived screen time changes, productivity, confounding factors, and emotional responses to smartphone use.

### Ethical considerations

This study was reviewed and approved by the California University of Science and Medicine Institutional Review Board (IRB Protocol #HS-2025-4) and determined to be exempt under 45 CFR 46. All data were de-identified prior to analysis to protect participant confidentiality. Both the researcher and statisticians were blinded to participant identities during analysis.

## Results

### Feasibility outcomes

Of the 61 participants enrolled, 51 completed both study phases, corresponding to a retention rate of 83.6%. Adherence to assigned display conditions was assessed via self-report and was generally high, with most participants reporting consistent use of the assigned setting during each study week. Qualitative responses further suggested acceptable usability, though some participants reported challenges with color-dependent applications.

### Group A: grayscale first sequence

Participants in Group A began the study in grayscale mode and then switched to the control condition in the second week. The mean screen time during the grayscale phase was 310.70 min per day (SD = 151.31), compared to 339.09 min per day in the control phase (SD = 210.86). The standard deviations were relatively high, suggesting expected substantial variability in individual responses.

A linear mixed-effects model with condition as a fixed effect and participant as a random intercept demonstrated a statistically significant reduction in screen time during grayscale compared to control [*β* = –28.39, SE = 11.53, t (324) = –2.46, *p* = 0.0143, 95% CI: −51.0 to −5.8]. Between-participant variance was estimated at 22,838 (SD = 151.1), with residual variance of 11,629 (SD = 107.8). Scaled residuals ranged from −3.09 to 3.21, indicating a satisfactory model fit (REML criterion = 4,336).

**Figure 2:**
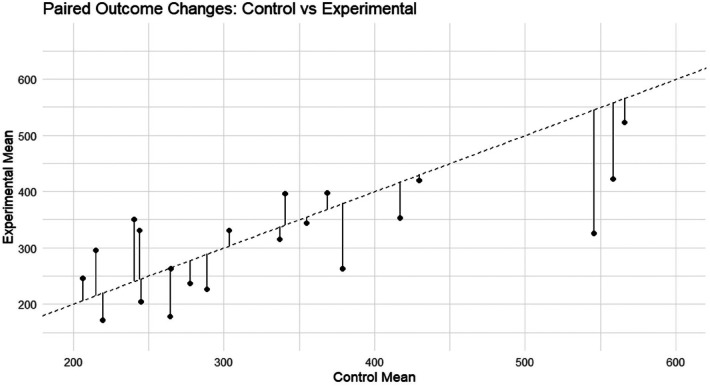
Individual paired screen time differences for group A.

Individual paired differences are illustrated in Figure 2.

### Group B: control first sequence

Participants in Group B began in the control condition before switching to grayscale. The mean screen time during the control phase was 355.18 min per day (SD = 132.81), compared to 326.74 min per day (SD = 133.67) during the grayscale phase.

A linear mixed-effects model again revealed a significant condition effect, with screen time reduced during grayscale [*β* = −28.44, SE = 9.94, t (337) = −2.86, *p* = 0.0045, 95% CI: −48.0 to −8.9]. Between-participant variance was estimated at 9,058 (SD = 95.17), and residual variance at 8,996 (SD = 94.85). Scaled residuals ranged from −2.78 to 3.85 (REML criterion = 4,401.4).

**Figure 3:**
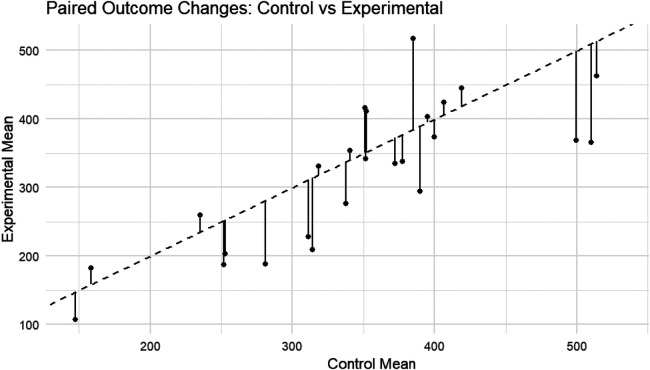
Individual paired screen time differences for group B.

Individual paired differences are illustrated in Figure 3.

### Combined condition analysis

To evaluate the overall effect of grayscale across all participants, screen time observations were pooled by condition (control vs. grayscale) across sequences.

**Figure 4:**
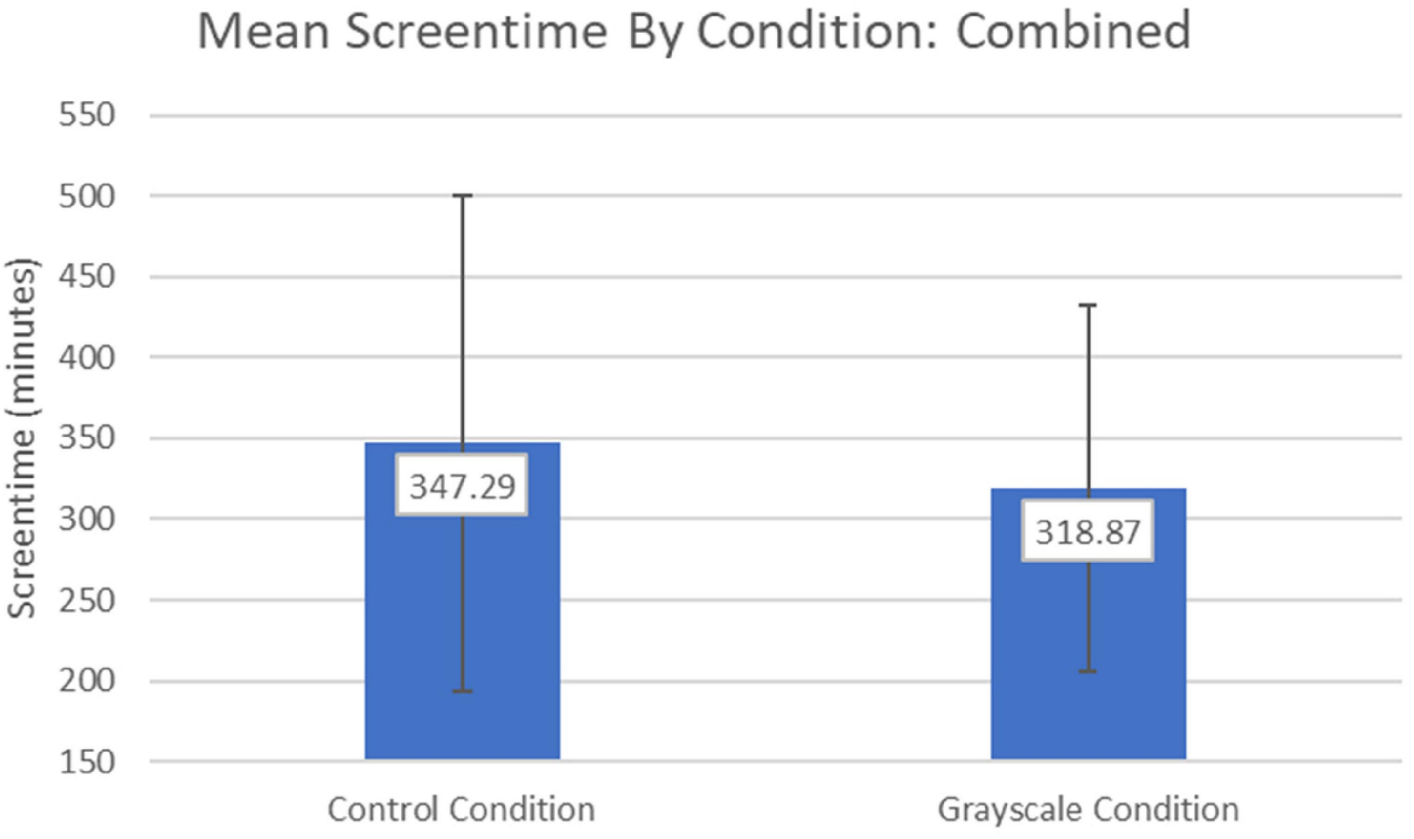
Mean daily screen time by condition across participants.

A comparison (Figure 4) illustrates substantial overlap between distributions, with the grayscale condition showing a lower average screen time (mean = 318.87, SD = 113.26) compared to the control condition (mean = 347.29, SD = 153.43). The grayscale condition was associated with an average reduction of approximately 28.42 min/day and this difference reached statistical significance in a paired-samples t-test (*t* = 2.3236, *p* = 0.0243, df = 50, SE = 12.229.

Exploratory analyses of self-reported productivity and sleep quality did not demonstrate consistent or statistically significant differences between grayscale and control conditions. These measures were collected using non-validated survey items and were not powered for formal comparison.

### Qualitative results

In addition to quantitative measures, participants were invited to provide free-text comments on their experiences with grayscale mode. Ten participants contributed qualitative responses. Thematic analysis identified several recurring domains, which are summarized below with representative examples.

#### Reduced screen time

One participant reported that grayscale mode decreased their smartphone use. However, substitution effects were noted, with screen time displaced to other devices:

“My screen time decreased, but honestly I think I spent that screen time on other devices. On gray(scale) I still used my phone as normal with the exception of watching shows/YouTube, but I ended up just using my laptop instead.”

#### Study and productivity impact

Two participants described how grayscale influenced their study or productivity-related phone use, particularly regarding educational applications:

“Anki, Zoom, and Teams are consistently my most used apps, which I consider productive.”

#### Confounding factors

Four participants identified external factors that inflated their measured screen time, including navigation, travel, or required academic platforms:

“Lots of this time was spent on maps, however still a good chunk on time wasting activities.”

“I drove to the Bay Area and back, and I had my phone on the entire time. This might be a confounding factor.”

#### Negative experiences and challenges

Two participants reported emotional reactions to their screen time, with comments reflecting embarrassment or frustration:

“The amount of time I spend on my phone is embarrassing.”

Overall, the qualitative data contextualize the quantitative results. While grayscale settings demonstrably altered screen time habits, the visual appeal of smartphones, external academic requirements, navigation, and substitution with other devices may have limited the ability for participants to gain the full benefit of the grayscale settings' impact.

## Discussion

In this cross-over feasibility trial, enabling grayscale display settings on smartphones was associated with a statistically significant reduction in daily screen time among medical students. This finding is consistent with prior grayscale intervention studies conducted in undergraduate and pharmacy student populations, which similarly demonstrated reductions in smartphone engagement. The crossover design allowed participants to serve as their own controls and demonstrated that the observed reduction was consistent regardless of intervention order, suggesting that neither novelty effects nor sequence effects meaningfully influenced the results. Together, these findings suggest that modifying visual salience within the digital environment can measurably influence engagement with smartphones during medical training.

From a health professions education perspective, these findings are best understood as an intervention that influences behavior indirectly through modification of the digital environment rather than through explicit restriction or instruction. Smartphones function as integral components of the contemporary learning environment, supporting activities such as spaced repetition, virtual conferencing, and clinical coordination. By reducing color saturation, grayscale mode alters perceptual salience and may reduce attentional capture by nonessential stimuli, thereby supporting more intentional engagement with digital tools. This aligns with prior work describing grayscale as a salience-reduction intervention that decreases the visual reward associated with smartphone use rather than restricting access. In this sense, grayscale mode may function as an attentional nudge that facilitates self-regulated learning rather than as a restrictive control on device use.

The absence of a meaningful sequence effect further supports this interpretation. If reductions in screen time were driven primarily by reactivity or awareness of monitoring, a diminishing effect across study phases might be expected. Instead, the persistence of the effect across sequences suggests that changes in visual interface characteristics exerted a stable influence on engagement patterns. This is consistent with prior work suggesting that perceptual features of interfaces can shape attention allocation and task persistence without requiring explicit behavioral mandates.

Qualitative findings provide important context for the quantitative results. Participants frequently described reduced habitual phone engagement while also noting substitution effects, usability challenges, and task-specific reliance on color-coded applications. These observations highlight that not all screen time is equivalent from an educational standpoint and that smartphones serve both productive and nonproductive functions within medical training. Rather than advocating for indiscriminate screen reduction, these findings suggest that educational interventions should consider how digital environments can be structured to support intentional, goal-directed use.

Several limitations should be considered when interpreting these findings. Screen time data were self-reported using built-in device tracking features without screenshot verification, which may have introduced measurement error due to social desirability bias, incomplete adherence to study instructions, unavailable tracking data, or variation in device settings (10). The short duration of the intervention limited assessment of longer-term adaptation or habituation effects, and blinding was not feasible due to the visible nature of the grayscale display. Additionally, external factors such as navigation requirements, videoconferencing, and travel may have inflated measured screen time independently of attentional engagement. These limitations are inherent to survey-based and self-monitoring designs and should be addressed in future studies using longer follow-up periods or complementary objective measures. Additionally, qualitative responses were provided by a limited subset of participants and may not fully represent the experiences or perceptions of the broader study population. As such, thematic findings should be interpreted as exploratory and contextual rather than comprehensive. Lastly, no washout period was included between study phases; although grayscale mode is unlikely to produce lasting carryover effects once color display is restored, residual behavioral adaptation cannot be entirely excluded.

Despite these limitations, this study contributes novel evidence to health professions education by demonstrating that a low-cost, readily accessible modification to the digital learning environment can influence engagement behaviors among medical students. **The consistency of these findings with prior grayscale intervention studies in undergraduate and pharmacy student populations suggests that interface-level modifications may represent a potentially transferable strategy for influencing digital engagement.** Future work should explore how interface-level modifications interact with individual differences in self-regulation and how such strategies may be integrated into broader educational design frameworks.

## Conclusion

This cross-over feasibility trial demonstrates that enabling grayscale display settings on smartphones is associated with a modest but consistent reduction in daily screen time among medical students. By modifying visual salience rather than restricting access to digital tools, grayscale mode represents a low-cost and readily accessible interface-level strategy that may influence engagement patterns in high-demand populations. **Participant qualitative feedback highlighted both perceived benefits and practical barriers, including substitution effects and reliance on color-dependent applications for navigation and educational tasks.** These findings should be interpreted as preliminary and hypothesis-generating with respect to screen time reduction. Larger studies with longer follow-up and objective behavioral measures are needed to determine the durability of effects and their relevance to digital behavior-change frameworks.

## Data Availability

The datasets presented in this study can be found in online repositories. The names of the repository/repositories and accession number(s) can be found in the article/Supplementary Material.
